# 4-(4-Pyrid­yl)pyridinium 3′,4,4′-tricarboxy­biphenyl-3-carboxyl­ate dihydrate

**DOI:** 10.1107/S1600536809050831

**Published:** 2009-12-09

**Authors:** Lu Han, Huan-Mian Luo, Qiu-Hui Meng, Yi-Fan Luo, Rong-Hua Zeng

**Affiliations:** a School of Chemistry and Environment, South China Normal University, Guangzhou 510006, People’s Republic of China; b School of Chemistry and Environment, South China Normal University, Guangzhou 510006, People’s Republic of China, and, South China Normal University, Key Laboratory of Technology of Electrochemical Energy Storage and Power Generation in Guangdong Universities, Guangzhou 510006, People’s Republic of China

## Abstract

In the title compound, C_10_H_9_N_2_^+^·C_16_H_9_O_8_^−^·2H_2_O, both the cation and anion possess crystallographically imposed centres of symmetry, causing the nitro­gen-bound H atom in the 4-(4-pyrid­yl)pyridinium cation and the acidic H atom of the carboxyl­ate groups at the 3 and 3′ positions in the anion to be disordered over two positions with equal occupancies. In the crystal packing, the cations, anions and water mol­ecules are connected by O—H⋯O, C—H⋯O and N—H⋯N hydrogen bonds, forming layers parallel to (2
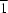
0). These layer are further connected into a three-dimensional supra­molecular network by O—H⋯O hydrogen bonds involving the water mol­ecules as H-atom donors and by weak π–π stacking inter­actions between neighbouring benzene and pyridine rings, with centroid–centroid distances of 3.756 (5) Å.

## Related literature

For related structures, see: Wang *et al.* (2006 ▶, 2007 ▶); Yang *et al.* (2007 ▶).
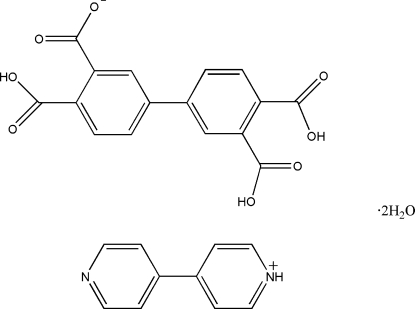


## Experimental

### 

#### Crystal data

C_10_H_9_N_2_^+^·C_16_H_9_O_8_^−^·2H_2_O
*M* *_r_* = 522.46
Triclinic, 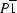

*a* = 7.1955 (8) Å
*b* = 8.935 (1) Å
*c* = 9.8847 (11) Å
α = 90.7920 (11)°
β = 106.4850 (13)°
γ = 107.1180 (15)°
*V* = 579.09 (11) Å^3^
*Z* = 1
Mo *K*α radiation
μ = 0.12 mm^−1^
*T* = 296 K
0.23 × 0.21 × 0.19 mm

#### Data collection

Bruker APEXII area-detector diffractometer
2990 measured reflections
2050 independent reflections
1818 reflections with *I* > 2σ(*I*)
*R*_int_ = 0.014

#### Refinement

*R*[*F* ^2^ > 2σ(*F* ^2^)] = 0.039
*wR*(*F* ^2^) = 0.112
*S* = 1.03
2050 reflections
180 parameters
3 restraints
H atoms treated by a mixture of independent and constrained refinement
Δρ_max_ = 0.32 e Å^−3^
Δρ_min_ = −0.25 e Å^−3^

### 

Data collection: *APEX2* (Bruker, 2005 ▶); cell refinement: *SAINT* (Bruker, 2005 ▶); data reduction: *SAINT*; program(s) used to solve structure: *SHELXS97* (Sheldrick, 2008 ▶); program(s) used to refine structure: *SHELXL97* (Sheldrick, 2008 ▶); molecular graphics: *SHELXTL* (Sheldrick, 2008 ▶); software used to prepare material for publication: *SHELXTL*.

## Supplementary Material

Crystal structure: contains datablocks I, global. DOI: 10.1107/S1600536809050831/rz2388sup1.cif

Structure factors: contains datablocks I. DOI: 10.1107/S1600536809050831/rz2388Isup2.hkl

Additional supplementary materials:  crystallographic information; 3D view; checkCIF report

## Figures and Tables

**Table 1:** Hydrogen-bond geometry (Å, °)

*D*—H⋯*A*	*D*—H	H⋯*A*	*D*⋯*A*	*D*—H⋯*A*
N1—H1*A*⋯N1^i^	0.92 (2)	1.89 (2)	2.814 (3)	177 (4)
O4—H4*A*⋯O1*W*	0.82	1.75	2.5657 (19)	176
O1*W*—H1*W*⋯O1^ii^	0.83	1.86	2.642 (2)	157
O1*W*—H2*W*⋯O3^iii^	0.84	2.03	2.841 (2)	165
O2—H2*A*⋯O2^iv^	0.91 (2)	1.54 (2)	2.446 (3)	173 (7)
C3—H3⋯O1^v^	0.93	2.54	3.270 (2)	135
C13—H13⋯O2^vi^	0.93	2.57	3.448 (2)	159
C9—H9⋯O2^vii^	0.93	2.36	3.246 (2)	160

Symmetry codes: (i) 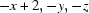; (ii) 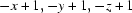; (iii) 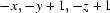; (iv) 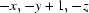; (v) 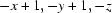; (vi) 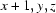; (vii) 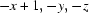.

**Fig. 1.**
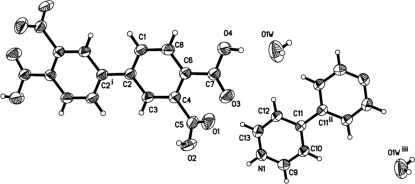
The molecular structure of the title compound showing the atomic-numbering scheme and 50% probability ellipsoids. Only one position of the disordered H atoms in the cation and anion is shown. Symmetry codes: (i) 1-x, 2-y, -z; (ii) 2-x, -y, 1-z; (iii) -x, 1-y, -z.

**Fig. 2.**
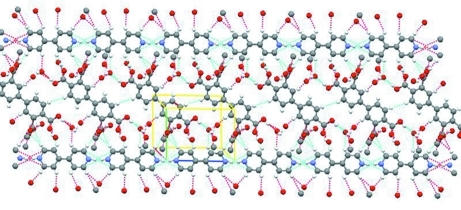
Partial packing diagram of the title compound showing the layered network formed by intermolecular O—H···O, C—H···O and N—H···N hydrogen bonds (dashed lines). Both positions of the disordered H atoms in the cation and anions are shown.

## supplementary crystallographic information

### Comment

In recent years, research on coordination polymers has made considerable progress in the fields of supramolecular chemistry and crystal engineering, because of their intriguing structural motifs and functional properties, such as molecular adsorption, magnetism, and luminescence. In general, hydrogen bonding interactions between ligands are specific and directional. In this context, biphenyl-3,3',4,4'-tetracarboxylic acid and 4,4'-bipyridine are excellent candidates for the construction of three-dimensional network motifs (Wang *et al.*, 2006; Wang *et al.*, 2007; Yang *et al.*, 2007). Recently, we obtained the title compound under hydrothermal conditions and report its crystal structure here.

The asymmetric unit of the title compound contains one half of a monoprotic 4-4'-bipyridinium cation, one half of a 3-3'-4-tricarboxybiphenyl-4'-carboxylato anion and a water molecule, with both cation and anion possessing crystallographically imposed centre of symmetry (Fig. 1). As a result, the H1A nitrogen-bound hydrogen atom in the cation and acidic H2A hydrogen atom in the anion are disordered over two positions with site occupancies of 0.5. Bond lengths and angles are unexceptional. In the crystal packing (Fig. 2), cations, anions and lattice water molecules are linked by intermolecular O—H···O, C—H···O and N—H···N hydrogen bonds (Table 1) into layers parallel to (2 1 0). The layers are further connected into a three-dimensional network by O—H···O hydrogen bonds involving the water molecules as H-donors and by weak π–π stacking interactions involving neighbouring benzene and pyridine rings, with centroid-to-centroid distances of 3.756 (5) Å.

### Experimental

A mixture of 4,4'-bipyridine (0.078 g, 0.5 mmol), 4-4'-dicarboxybiphenyl-3-3'dicarboxylic acid (0.165 g; 0.5 mmol), water (10 mL) was stirred vigorously for 30 min and then sealed in a Teflon-lined stainless-steel autoclave (25 mL capacity). The autoclave was heated and maintained at 433K for 3 days, and then cooled to room temperature at 5Kh^-1^ to obtain colourless block crystals suitable for X-ray analysis.

### Refinement

Water H atoms were located in a difference Fourier map and refined with the O–H distance restrained to 0.84 Å and with *U*_iso_(H) = 1.5 *U*_eq_(O). The H atom bound to the N1 nitrogen atom in the cation and the carboxylic H atoms were refined with distance restraints of N–H = 0.90 Å and O–H = 0.90 Å, respectively. The H1A and H2A hydrogen atoms are disordered over two centrosymmetrically related positions and were therefore refined with site occupancies of 0.5. All other H atoms were placed at calculated positions and treated as riding on the parent atoms, with C–H = 0.93Å, and with *U*_iso_(H) = 1.2 *U*_eq_(C).

### Crystal data

**Table:**

C_10_H_9_N_2_^+^·C_16_H_9_O_8_^−^·2H_2_O	*Z* = 1
*M**_r_* = 522.46	*F*(000) = 272
Triclinic, *P*1	*D*_x_ = 1.498 Mg m^−^^3^
Hall symbol: -P 1	Mo *K*α radiation, λ = 0.71073 Å
*a* = 7.1955 (8) Å	Cell parameters from 2061 reflections
*b* = 8.935 (1) Å	θ = 2.4–28.2°
*c* = 9.8847 (11) Å	µ = 0.12 mm^−^^1^
α = 90.7920 (11)°	*T* = 296 K
β = 106.4850 (13)°	Block, colourless
γ = 107.1180 (15)°	0.23 × 0.21 × 0.19 mm
*V* = 579.09 (11) Å^3^	

### Data collection

**Table:**

Bruker APEXII area-detector diffractometer	1818 reflections with *I* > 2σ(*I*)
Radiation source: fine-focus sealed tube	*R*_int_ = 0.014
graphite	θ_max_ = 25.2°, θ_min_ = 2.2°
φ and ω scan	*h* = −7→8
2990 measured reflections	*k* = −10→9
2050 independent reflections	*l* = −11→11

### Refinement

**Table:**

Refinement on *F*^2^	Secondary atom site location: difference Fourier map
Least-squares matrix: full	Hydrogen site location: inferred from neighbouring sites
*R*[*F*^2^ > 2σ(*F*^2^)] = 0.039	H atoms treated by a mixture of independent and constrained refinement
*wR*(*F*^2^) = 0.112	*w* = 1/[σ^2^(*F*_o_^2^) + (0.0562*P*)^2^ + 0.2257*P*] where *P* = (*F*_o_^2^ + 2*F*_c_^2^)/3
*S* = 1.03	(Δ/σ)_max_ < 0.001
2050 reflections	Δρ_max_ = 0.32 e Å^−^^3^
180 parameters	Δρ_min_ = −0.25 e Å^−^^3^
3 restraints	Extinction correction: *SHELXL97* (Sheldrick, 2008), Fc^*^=kFc[1+0.001xFc^2^λ^3^/sin(2θ)]^-1/4^
Primary atom site location: structure-invariant direct methods	Extinction coefficient: 0.071 (9)

### Special details

**Table:**

Geometry. All esds (except the esd in the dihedral angle between two l.s. planes) are estimated using the full covariance matrix. The cell esds are taken into account individually in the estimation of esds in distances, angles and torsion angles; correlations between esds in cell parameters are only used when they are defined by crystal symmetry. An approximate (isotropic) treatment of cell esds is used for estimating esds involving l.s. planes.
Refinement. Refinement of F^2^ against ALL reflections. The weighted R-factor wR and goodness of fit S are based on F^2^, conventional R-factors R are based on F, with F set to zero for negative F^2^. The threshold expression of F^2^ > 2sigma(F^2^) is used only for calculating R-factors(gt) etc. and is not relevant to the choice of reflections for refinement. R-factors based on F^2^ are statistically about twice as large as those based on F, and R- factors based on ALL data will be even larger.

### Fractional atomic coordinates and isotropic or equivalent isotropic displacement parameters (Å^2^)

**Table:**

	*x*	*y*	*z*	*U*_iso_*/*U*_eq_	Occ. (<1)
C1	0.4881 (3)	1.01415 (19)	0.18864 (18)	0.0405 (4)	
H1	0.5318	1.1230	0.2095	0.049*	
C2	0.4740 (2)	0.94960 (17)	0.05542 (16)	0.0321 (4)	
C3	0.4103 (3)	0.78512 (18)	0.03012 (17)	0.0361 (4)	
H3	0.4008	0.7393	−0.0576	0.043*	
C4	0.3611 (2)	0.68843 (17)	0.13133 (17)	0.0333 (4)	
C5	0.3102 (3)	0.51258 (19)	0.09704 (18)	0.0397 (4)	
C6	0.3725 (2)	0.75584 (18)	0.26291 (16)	0.0344 (4)	
C7	0.3003 (3)	0.65386 (19)	0.36673 (18)	0.0389 (4)	
C8	0.4384 (3)	0.91911 (19)	0.28990 (18)	0.0407 (4)	
H8	0.4490	0.9650	0.3779	0.049*	
C9	0.9236 (3)	−0.1318 (2)	0.19327 (19)	0.0447 (4)	
H9	0.8726	−0.2254	0.1336	0.054*	
C10	0.9232 (3)	−0.13691 (19)	0.33220 (18)	0.0412 (4)	
H10	0.8722	−0.2331	0.3647	0.049*	
C11	0.9987 (2)	0.00096 (18)	0.42445 (17)	0.0351 (4)	
C12	1.0714 (3)	0.1400 (2)	0.3674 (2)	0.0490 (5)	
H12	1.1234	0.2355	0.4243	0.059*	
C13	1.0666 (3)	0.1367 (2)	0.2273 (2)	0.0524 (5)	
H13	1.1151	0.2311	0.1914	0.063*	
N1	0.9947 (2)	0.00297 (17)	0.14119 (15)	0.0428 (4)	
H1A	0.997 (7)	0.004 (5)	0.049 (2)	0.051*	0.50
O1	0.4404 (2)	0.44851 (15)	0.14281 (16)	0.0572 (4)	
O3	0.1949 (2)	0.51807 (15)	0.33352 (14)	0.0596 (4)	
O4	0.3524 (3)	0.72538 (16)	0.49433 (14)	0.0647 (5)	
H4A	0.3092	0.6627	0.5465	0.097*	
O1W	0.2145 (3)	0.5413 (2)	0.66452 (17)	0.0799 (6)	
H1W	0.3004	0.5290	0.7356	0.120*	
H2W	0.1031	0.5241	0.6817	0.120*	
O2	0.1336 (2)	0.43770 (14)	0.01377 (15)	0.0520 (4)	
H2A	0.040 (8)	0.490 (8)	0.001 (8)	0.078*	0.50

### Atomic displacement parameters (Å^2^)

**Table:**

	*U*^11^	*U*^22^	*U*^33^	*U*^12^	*U*^13^	*U*^23^
C1	0.0520 (10)	0.0255 (8)	0.0388 (9)	0.0025 (7)	0.0159 (8)	−0.0015 (7)
C2	0.0325 (8)	0.0276 (8)	0.0339 (8)	0.0060 (6)	0.0100 (6)	0.0017 (6)
C3	0.0467 (9)	0.0279 (8)	0.0327 (8)	0.0081 (7)	0.0141 (7)	−0.0006 (6)
C4	0.0346 (8)	0.0277 (8)	0.0352 (8)	0.0066 (6)	0.0102 (6)	0.0025 (6)
C5	0.0549 (10)	0.0293 (8)	0.0396 (9)	0.0115 (8)	0.0231 (8)	0.0057 (7)
C6	0.0353 (8)	0.0308 (8)	0.0339 (8)	0.0054 (6)	0.0104 (7)	0.0027 (6)
C7	0.0447 (9)	0.0324 (8)	0.0376 (9)	0.0063 (7)	0.0152 (7)	0.0023 (7)
C8	0.0526 (10)	0.0325 (8)	0.0323 (9)	0.0041 (7)	0.0153 (7)	−0.0022 (7)
C9	0.0530 (11)	0.0371 (9)	0.0421 (10)	0.0122 (8)	0.0135 (8)	−0.0016 (7)
C10	0.0513 (10)	0.0285 (8)	0.0431 (10)	0.0093 (7)	0.0163 (8)	0.0035 (7)
C11	0.0373 (8)	0.0286 (8)	0.0391 (9)	0.0092 (6)	0.0119 (7)	0.0039 (6)
C12	0.0691 (13)	0.0293 (9)	0.0425 (10)	0.0041 (8)	0.0190 (9)	0.0022 (7)
C13	0.0714 (13)	0.0379 (10)	0.0451 (10)	0.0079 (9)	0.0226 (9)	0.0093 (8)
N1	0.0516 (9)	0.0454 (8)	0.0332 (8)	0.0152 (7)	0.0151 (7)	0.0064 (6)
O1	0.0725 (10)	0.0442 (7)	0.0666 (9)	0.0307 (7)	0.0254 (7)	0.0142 (6)
O3	0.0830 (10)	0.0355 (7)	0.0522 (8)	−0.0054 (7)	0.0330 (7)	0.0011 (6)
O4	0.0976 (11)	0.0443 (8)	0.0368 (7)	−0.0065 (7)	0.0266 (7)	0.0019 (6)
O1W	0.0693 (10)	0.0986 (13)	0.0630 (10)	0.0071 (9)	0.0252 (8)	0.0340 (9)
O2	0.0599 (9)	0.0273 (6)	0.0601 (8)	0.0046 (6)	0.0146 (7)	−0.0086 (6)

### Geometric parameters (Å, °)

**Table:**

C1—C8	1.378 (2)	C9—N1	1.334 (2)
C1—C2	1.395 (2)	C9—C10	1.375 (3)
C1—H1	0.9300	C9—H9	0.9300
C2—C3	1.400 (2)	C10—C11	1.392 (2)
C2—C2^i^	1.488 (3)	C10—H10	0.9300
C3—C4	1.385 (2)	C11—C12	1.392 (2)
C3—H3	0.9300	C11—C11^ii^	1.489 (3)
C4—C6	1.397 (2)	C12—C13	1.375 (3)
C4—C5	1.514 (2)	C12—H12	0.9300
C5—O1	1.222 (2)	C13—N1	1.334 (2)
C5—O2	1.277 (2)	C13—H13	0.9300
C6—C8	1.391 (2)	N1—H1A	0.921 (18)
C6—C7	1.484 (2)	O4—H4A	0.8200
C7—O3	1.209 (2)	O1W—H1W	0.8263
C7—O4	1.307 (2)	O1W—H2W	0.8370
C8—H8	0.9300	O2—H2A	0.911 (19)
			
C8—C1—C2	121.02 (15)	C6—C8—H8	119.4
C8—C1—H1	119.5	N1—C9—C10	122.24 (16)
C2—C1—H1	119.5	N1—C9—H9	118.9
C1—C2—C3	117.26 (15)	C10—C9—H9	118.9
C1—C2—C2^i^	121.74 (17)	C9—C10—C11	120.39 (16)
C3—C2—C2^i^	121.00 (17)	C9—C10—H10	119.8
C4—C3—C2	122.24 (15)	C11—C10—H10	119.8
C4—C3—H3	118.9	C10—C11—C12	116.23 (16)
C2—C3—H3	118.9	C10—C11—C11^ii^	121.63 (18)
C3—C4—C6	119.43 (14)	C12—C11—C11^ii^	122.14 (18)
C3—C4—C5	117.83 (14)	C13—C12—C11	120.36 (16)
C6—C4—C5	122.62 (14)	C13—C12—H12	119.8
O1—C5—O2	122.14 (16)	C11—C12—H12	119.8
O1—C5—C4	119.75 (16)	N1—C13—C12	122.31 (17)
O2—C5—C4	117.99 (15)	N1—C13—H13	118.8
C8—C6—C4	118.80 (15)	C12—C13—H13	118.8
C8—C6—C7	121.15 (14)	C13—N1—C9	118.47 (15)
C4—C6—C7	119.90 (14)	C13—N1—H1A	120 (3)
O3—C7—O4	123.27 (16)	C9—N1—H1A	121 (3)
O3—C7—C6	122.33 (15)	C7—O4—H4A	109.5
O4—C7—C6	114.33 (14)	H1W—O1W—H2W	109.1
C1—C8—C6	121.24 (15)	C5—O2—H2A	115 (5)
C1—C8—H8	119.4		

Symmetry codes: (i) −*x*+1, −*y*+2, −*z*; (ii) −*x*+2, −*y*, −*z*+1.

### Hydrogen-bond geometry (Å, °)

**Table:**

*D*—H···*A*	*D*—H	H···*A*	*D*···*A*	*D*—H···*A*
N1—H1A···N1^iii^	0.92 (2)	1.89 (2)	2.814 (3)	177 (4)
O4—H4A···O1W	0.82	1.75	2.5657 (19)	176
O1W—H1W···O1^iv^	0.83	1.86	2.642 (2)	157
O1W—H2W···O3^v^	0.84	2.03	2.841 (2)	165
O2—H2A···O2^vi^	0.91 (2)	1.54 (2)	2.446 (3)	173 (7)
C3—H3···O1^vii^	0.93	2.54	3.270 (2)	135
C13—H13···O2^viii^	0.93	2.57	3.448 (2)	159
C9—H9···O2^ix^	0.93	2.36	3.246 (2)	160

Symmetry codes: (iii) −*x*+2, −*y*, −*z*; (iv) −*x*+1, −*y*+1, −*z*+1; (v) −*x*, −*y*+1, −*z*+1; (vi) −*x*, −*y*+1, −*z*; (vii) −*x*+1, −*y*+1, −*z*; (viii) *x*+1, *y*, *z*; (ix) −*x*+1, −*y*, −*z*.
